# Valorisation of cheese whey into single-cell protein by Lactococcus garvieae: dual waste nitrogen supplementation and uncertainty-informed process evaluation

**DOI:** 10.1007/s11274-026-04911-3

**Published:** 2026-04-21

**Authors:** Halil İbrahim Uzun, Harun Önlü

**Affiliations:** 1 Department of Food Engineering, Faculty of Engineering and Architecture, Muş Alparslan University, Muş, Türkiye; 2 Department of Food Processing, Vocational School of Technical Sciences, Muş Alparslan University, Muş, Türkiye

**Keywords:** Single-cell protein, Cheese whey; waste valorisation, Lactic acid bacteria, Uncertainty analysis

## Abstract

Growing global protein demand necessitates resource-efficient alternatives to conventional production systems. This study developed a waste-based bioprocess framework for converting cheese whey (CW) into single-cell protein (SCP) using lactic acid bacteria within a dual waste-valorisation configuration integrating CW and expired chickpea flour–derived extract. Seven LAB strains were screened in pre-treated CW, and Lactococcus garvieae was selected based on its highest biomass formation in untreated whey (0.441 ± 0.024 g/L). As CW was nitrogen-limited, supplementation with water-soluble legume extracts significantly enhanced SCP production, with chickpea extract (CE) yielding 1.560 ± 0.120 g/L at 10 g/L. Among the tested carbon sources, sucrose supported the highest biomass concentration (2.110 ± 0.105 g/L). Under the selected cultivation conditions (pH 7.0, 37 °C), SCP reached 2.314 ± 0.090 g/L. Although the highest observed biomass concentration occurred at 72 h (2.663 ± 0.154 g/L), volumetric productivity peaked at 24 h (0.096 g/L·h), which was therefore selected as the operational incubation time based on the productivity–time trade-off. Second-order polynomial modelling combined with Delta Method–based uncertainty estimation enabled quantitative differentiation between model-derived stationary points and experimentally justified operating conditions. The integration of expired legume flour as a waste-derived nitrogen source, together with uncertainty-aware response modelling within an OFAT framework, establishes a dual waste-valorisation strategy and a transparent approach for statistically supported process window identification under laboratory-scale constraints. The resulting SCP biomass contained 56.37 ± 2.75% protein, 2.87 ± 0.34% lipids, and 10.55 ± 0.68% ash.

**Supplementary Information:**

The online version contains supplementary material available at 10.1007/s11274-026-04911-3.

## Introduction

Population growth is driving a rapid increase in global protein demand, placing growing pressure on the sustainability of conventional protein production systems. The expansion of agricultural production is increasingly constrained by land availability, water resources, and energy inputs, limiting the capacity of existing systems to meet future demand (Fasihi et al. 2025; Yang et al. 2025).

Single-cell protein (SCP) derived from microorganisms has emerged as a promising alternative protein platform due to its rapid production kinetics and high protein density (Chamodi et al. 2025; Suman et al. 2015). Owing to the short generation times of microorganisms, SCP production enables shorter production cycles and higher volumetric outputs compared to agricultural protein sources (Nasseri et al. 2011). Nevertheless, the industrial applicability of SCP production largely depends on key performance indicators such as biomass yield and process efficiency.

Bacterial platforms offer important advantages for SCP production due to their high protein content and rapid growth kinetics. However, processes involving gram-negative bacteria are associated with endotoxin contamination, which necessitates additional purification steps, particularly for food and feed applications, thereby increasing biosafety risks and production costs (Zhuang et al. 2024). In this context, lactic acid bacteria (LAB) comprise a group of fermentative microorganisms widely applied in food systems and traditional fermentations (Liu et al. 2021; Plavec and Berlec 2020). Despite the growing number of studies investigating SCP production using LAB, biomass accumulation is rarely positioned as the primary process objective. Instead, LAB-based fermentations are predominantly structured around metabolite production, and cell mass is typically treated as a secondary performance indicator (Ritala et al. 2017; Agregán-Pérez et al. 2021; Singha et al. 2021). In industrial practice, LAB are generally selected and developed for the synthesis of lactic acid (LA), exopolysaccharides, bacteriocins, vitamins, and flavor-active compounds, which constitute the principal commercial outputs of these systems (Li et al. 2025; Tang et al. 2023; Wang et al. 2021; Zeidan et al. 2017). Accordingly, LAB are not typically considered conventional platforms for high-density SCP production, as carbon flux in fermentative metabolism is largely directed toward metabolite synthesis rather than maximal biomass accumulation. Against this background, the selection of substrate becomes a critical determinant of whether LAB-based systems can be reoriented from metabolite-centered processes toward biomass-focused applications.

At the same time, the industrial feasibility of SCP production remains strongly dependent on substrate cost, biomass yield, and process configuration, prompting extensive investigation of waste streams as alternative substrates (Kaur and Chavan 2022; Khan et al. 2024; Thiviya et al. 2022). However, many existing studies assess process performance based on isolated parameters, which may limit the transferability of laboratory-scale findings to industrial conditions (Ye et al., 2024).

Cheese whey (CW) is a high-volume by-product of the dairy industry and poses environmental risks when discharged without treatment (Malos et al. 2025; Monkoondee et al. 2016). Owing to its high lactose content and dissolved nutrients, CW represents a suitable carbon source for microbial growth; however, when used as a sole substrate, imbalances in the carbon-to-nitrogen (C/N) ratio may constrain biomass formation (Delgado-Macuil et al. 2025; Paraskevopoulou et al. 2003). Accordingly, the integration of expired legume stocks and flours as alternative nitrogen sources, instead of costly commercial inputs, offers a strategic approach to enhance biomass yield while simultaneously recovering food waste.

LAB-based fermentation of CW has predominantly been investigated for the production of value-added end products. Within this body of work, SCP has largely been treated as a secondary output, and limited attention has been given to the systematic evaluation of biomass yield as a primary objective (Agregán-Pérez et al. 2021; Delgado-Macuil et al. 2025; Monkoondee et al. 2016). Moreover, studies that comprehensively examine the interactions between key fermentation parameters—such as pH, carbon and nitrogen sources—and biomass accumulation using kinetic and statistical frameworks remain scarce (Amini et al. 2025; Yang et al. 2025). Collectively, these limitations indicate the absence of (i) strain-level evaluation of LAB for biomass-oriented CW valorization, (ii) integration of secondary waste-derived nitrogen streams, and (iii) uncertainty-aware analytical frameworks capable of distinguishing model-derived stationary points from process-relevant operational conditions.

To address these limitations, this study presents a statistically grounded bioprocess framework for the systematic assessment of SCP production from CW through controlled evaluation of pH, temperature, fermentation time, and carbon source. A comparative strain-level screening was performed to identify taxa capable of redirecting carbon flux toward biomass accumulation under nitrogen-constrained whey conditions. The CW medium was enriched with waste-derived legume extracts within a dual waste valorization strategy aimed at reducing nitrogen source costs. Process responses were described using second-degree polynomial regression and the Delta Method (DM), enabling quantitative uncertainty assessment and a transparent distinction between model-derived stationary points and operationally selected conditions.

## Materials & methods

### Microorganisms and inoculum preparation

In this study, seven LAB strains—*Enterococcus faecium* MH5, *Lactobacillus sakei* MH1, *Lactococcus garvieae* MH3, *Lactococcus lactis subsp. cremoris* MH13, *Lactococcus lactis* MH31, *Weissella confusa* MH26, *and Lactobacillus plantarum* TOS9—were employed to evaluate their potential for SCP production. The strains were isolated from various traditional food matrices (Onur and Önlü 2021, 2024; Önlü and Osmanağaoğlu 2025).

For stock culture activation, frozen cultures were aseptically transferred into 10 mL of MRS broth (De Man, Rogosa and Sharpe; Biolife, Italy) and incubated at 37 °C for 24 h using a temperature-controlled incubator (WiseCube WIG 105, Daihan Scientific, South Korea). To standardize metabolic activity and minimize the lag phase, actively growing cultures were subcultured into fresh MRS medium at a ratio of 1:100 (v/v). Following the second passage, cell density was determined by serial dilution in sterile peptone water (0.1%, w/v; Sigma-Aldrich, Germany) and plate counting on solidified MRS medium prepared by supplementing MRS broth (De Man, Rogosa and Sharpe; Biolife, Italy) with bacteriological agar (ISOLAB, Laborgeräte GmbH, Germany), after incubation at 37 °C for 24 h. For all experiments, the initial inoculum concentration was standardized to approximately 1 × 10⁸ CFU/mL.

### Substrate pre-treatment and characterization

CW obtained from a local dairy facility in Muş, Turkiye was used as the fermentation substrate. The CW, which had already undergone thermal treatment during the curd production process at the facility, was subjected to preliminary laboratory-scale clarification to remove coarse particles and residual solids. For this purpose, samples were autoclaved at 121 °C for 15 min using a laboratory autoclave (Steamart OT-40 L, Nüve, Turkiye) to facilitate the precipitation of remaining residues, rapidly cooled to 4 °C, and subsequently clarified by centrifugation at 6500 × g for 10 min in a refrigerated centrifuge (NF800R, Nüve, Turkiye). The pH of the resulting supernatant was adjusted to 6.7 using 5 N NaOH (ISOLAB Laborgeräte GmbH, Germany), as monitored with a portable pH/temperature meter (MW102 PRO+, Milwaukee Instruments, Romania), and re-verified before the final sterilisation step. Post-sterilisation pH measurements confirmed deviations of no more than ± 0.1 units from the target value, consistent with the removal of major pH-reactive components during the preceding thermal precipitation and centrifugation steps. The prepared basal medium was sterilized at 121 °C for 15 min prior to inoculation using the same autoclave (Alkan et al., 2019). To characterize the initial nutrient composition of the substrate, chemical oxygen demand (COD) was determined using commercial digestion tubes (Tubetest^®^57, 1.199.15000, Matriks, Turkiye) processed in a thermoreactor (Matriks, Turkiye) and measured spectrophotometrically using a UV–Vis spectrophotometer (UV-1800, Shimadzu, Japan) (Lipps et al. 2023). Total nitrogen (TN) was determined by the Kjeldahl method after digestion in a Kjeldahl digestion unit (DKL Heating Digester, VELP Scientifica, Italy) (Lipps et al. 2023). Total phosphorus (TP) was quantified spectrophotometrically according to Standard Methods using the same UV–Vis spectrophotometer (Lipps et al. 2023).

### Preparation of legume-based nitrogen supplements for nutrient fortification

Water-soluble extracts derived from legume sources excluded from the human food supply chain due to the expiration of their shelf life (chickpea, green lentil, and red lentil) were used to enrich the organic nitrogen and, partially, the carbon content of the CW-based fermentation substrate. The legume extracts were prepared following water-based extraction protocols described in the literature for the recovery of water-soluble protein and carbohydrate fractions from legume seeds (Jarpa-Parra et al. 2014; Siqueira et al. 2025). A similar extraction approach was previously applied in our preliminary study which is further expanded and systematically evaluated in the present work (Uzun and Önlü 2024). Briefly, ground legume flours were mixed with deionized water at a concentration of 10% (w/v) and extracted overnight (≈ 16 h) at room temperature in an orbital shaker (OS200, Allsheng, China) to avoid thermal denaturation. The resulting suspensions were centrifuged at 5000 × g for 30 min to separate insoluble fractions, and the supernatants were dried at 60 °C for 24 h in a drying oven (WiseVen WOF-155, Daihan Scientific, South Korea). The dried extracts were subsequently ground using a mortar, passed through a 150 μm sieve (Jeotest, Turkiye), homogenized, and stored under sterile conditions. The prepared water-soluble legume extracts were added to the fermentation substrate as nitrogen supplements to support the growth of *L. garvieae* in the CW-based medium.

### Experimental design and fermentation processes

#### Strain screening and biomass production

In the initial stage of the study, the SCP production performance of seven LAB strains was comparatively evaluated in pre-treated CW-based substrate. Fermentations were conducted in 100 mL glass bottles containing 90 mL of sterile CW. The substrate was inoculated with actively growing cultures at an initial density of 1 × 10⁸ CFU/mL, and the cultures were incubated under static conditions at 37 °C for 24 h (Zhang et al. 2016). The screening was conducted under identical cultivation parameters to enable direct comparison of biomass-forming capacity across strains at this preliminary stage. Following incubation, the strain exhibiting the highest biomass yield under these standardized conditions was selected as the model organism for subsequent process evaluation. This strain was identified as Lactococcus garvieae MH3 and is deposited in the NCBI BioSample database (Accession: SAMN24475444) (NCBI, 2021).

#### Sequential evaluation of culture medium composition and physical process conditions

The composition of the CW-based fermentation medium was sequentially evaluated using a One-Factor-at-a-Time (OFAT) experimental design to identify key nutritional variables influencing SCP yield. This approach was applied as an initial screening strategy while maintaining a feasible experimental scope. As CW composition may vary with seasonal conditions, geographical origin, and processing practices and represents a chemically heterogeneous substrate, the results obtained here are specific to the investigated system and operating conditions. The experimental evaluation consisted of the following sequential steps: (i) Nitrogen source screening, in which different plant-based extracts (chickpea extract (CE), green lentil extract (GLE), and red lentil extract (RLE) were added to the CW-based substrate at 10 g/L and their effects on biomass yield were evaluated; (ii) Nitrogen level assessment, in which CE was supplemented at 10, 15, 20, and 30 g/L to evaluate the influence of nitrogen loading on SCP production; (iii) Carbon source screening, in which glucose, fructose, sucrose, and lactose (Sigma-Aldrich, Germany) were individually supplemented at 50 g/L (w/v) under the selected nitrogen conditions; (iv) Carbon level assessment, in which the selected carbon source was supplemented at 50, 100, and 150 g/L to examine system response; (v) pH assessment, in which initial pH values of 5.0, 6.0, 7.0, and 8.0 were tested, covering the commonly reported growth range of LAB and spanning mildly acidic to near-alkaline conditions typically evaluated in LAB cultivation studies (vi) Temperature assessment, conducted at 30, 37, 40, and 42 °C, covering the mesophilic growth range of *L. garvieae*, with 37 °C corresponding to the reported optimal growth temperature and 42 °C representing the upper physiological growth limit described in previous studies; and (vii) Fermentation time assessment, evaluated at 24, 36, 48, and 72 h (Gibello et al. 2016; Hussein et al. 2015).

### Biomass harvesting and analytical methods

At the end of the fermentation period, microbial biomass was harvested using a refrigerated centrifuge at 5000 × g for 20 min at 4 °C (NF800R, Nüve, Turkiye). The resulting cell pellets were washed twice with sterile 0.9% (w/v) NaCl (ISOLAB, Laborgeräte GmbH, Germany) solution to remove residual fermentation medium and subsequently centrifuged. The washed pellets were dried at room temperature until constant weight was achieved (Kunasundari et al. 2013), and SCP yield was determined gravimetrically and expressed as g/L. The crude protein content of the harvested biomass was determined using the Kjeldahl method, with total nitrogen converted to protein using a conversion factor of 6.25 (Horwitz 1975; ISO 1871, 2009). Lipid content was quantified gravimetrically following acid hydrolysis according to ISO 1443 (1973). Total carbohydrate content was determined using the phenol–sulfuric acid method and expressed as glucose equivalents (DuBois et al. 1956). Crude ash content was measured gravimetrically after incineration of samples in a high-temperature muffle furnace (Mikrolab MKF108, Turkiye).

### Statistical analysis

All experiments were conducted in triplicate (*n* = 3), and the results are expressed as mean ± standard deviation (SD). Prior to statistical analysis, the data sets were assessed for compliance with parametric test assumptions using the Shapiro–Wilk test for normality and Levene’s test for homogeneity of variances (*p* > 0.05). Differences among experimental groups were evaluated using one-way analysis of variance (ANOVA), and statistically significant differences (*p* < 0.05) were further analyzed using Tukey’s honestly significant difference (HSD) post hoc test. All statistical analyses were performed using IBM SPSS Statistics version 27.0 (IBM Corp., Armonk, NY, USA).

The experimental design and general statistical approach applied in this study are partially based on previous work reported in Uzun 2025.

#### Process modelling and uncertainty analysis of model-derived response maxima

An advanced computational modelling framework was applied to characterise the non-linear relationships between fermentation parameters and SCP production and to quantify the uncertainty of model-derived response maxima under industrially relevant constraints. The experimental data were fitted to second-order (quadratic) polynomial regression models using the Python programming environment (v3.12.12) with the SciPy (v1.16.3) and NumPy (v2.0.2) libraries.

The general form of the model was:

1 $$\:\begin{array}{c}Y={\beta\:}_0+{\beta\:}_1x+{\beta\:}_2x^2\end{array}$$

where Y represents the predicted SCP concentration (g/L), x denotes the independent variable (e.g., substrate concentration, pH, temperature, or time), and β₀, β₁, and β₂ are the intercept, linear, and quadratic coefficients, respectively (Montgomery, 2017; Myers et al. 2016). Model parameters were estimated by ordinary least squares (OLS) minimisation using individual replicate observations (*n* = 3 per condition) and implemented via the SciPy curve_fit function. The statistical reliability of the estimated response maxima (X*, Y*) was evaluated using the DM (Cox 2005). Based on the variance–covariance matrix of the regression coefficients, standard errors (SE) and 95% confidence intervals (CI) were calculated to quantify model uncertainty. The goodness of fit was assessed using the coefficient of determination (R²) and adjusted R²:

2 $$\:\begin{array}{c}R^2=1-\frac{{\mathrm{S}\mathrm{S}}_{\mathrm{r}\mathrm{e}\mathrm{s}}}{{\mathrm{S}\mathrm{S}}_{\mathrm{t}\mathrm{o}\mathrm{t}}}\end{array}$$

3 $$\:\begin{array}{c}R_{\mathrm{a}\mathrm{d}\mathrm{j}}^2=1-\frac{\left[\left(1-R^2\right)\left(n-1\right)\right]}{\left(n-p-1\right)}\end{array}$$

where $\:{\mathrm{S}\mathrm{S}}_{\mathrm{r}\mathrm{e}\mathrm{s}}$ and $\:{\mathrm{S}\mathrm{S}}_{\mathrm{t}\mathrm{o}\mathrm{t}}$ denote the residual and total sums of squares, n is the number of observations, and p is the number of estimated parameters. Prediction accuracy was further evaluated using the root mean square error.

$\:\mathrm{R}\mathrm{M}\mathrm{S}\mathrm{E}\:=\:\sqrt{\frac{{\mathrm{S}\mathrm{S}}_{\mathrm{r}\mathrm{e}\mathrm{s}}}{\mathrm{n}-\mathrm{p}}}$. Residual normality was verified using the Shapiro–Wilk test (α = 0.05) to confirm compliance with OLS assumptions.

For models exhibiting a concave response (β₂ < 0), the stationary point of the quadratic function (x*) was derived analytically from the first derivative:

4 $$\:\begin{array}{c}x^\mathrm{*}=-\frac{{\beta\:}_1}{2{\beta\:}_2}\end{array}$$

The uncertainty associated with the estimated stationary point was quantified using the DM (Cox 2005). Based on the variance–covariance matrix (Σ) of the regression coefficients, the variance of x* was calculated as:

5 $$\:\begin{aligned}&\\&Var\left(x^\mathrm{*}\right)=\left[\frac1{2{\beta\:}_2}\right]^2\cdot\:Var\left({\beta\:}_1\right)+\left[\frac{{\beta\:}_1}{2{\beta\:}_2^2}\right]^2\cdot&\\&\:Var\left({\beta\:}_2\right)-2\cdot\:\left[\frac1{2{\beta\:}_2}\right]\cdot\:\left[\frac{{\beta\:}_1}{2{\beta\:}_2^2}\right]\cdot\:Cov({\beta\:}_1,{\beta\:}_2)\end{aligned}$$

The standard error was obtained as SE(x*) = √Var(x*), and 95% CI were constructed as:

6 $$X^\ast\pm t_{0.025,v}\cdot\;SE\left(x^\ast\right)$$

where t₀.₀₂₅,_v_ is the critical value of the t-distribution with ν = n − p degrees of freedom. The predicted maximum SCP yield at the model-derived stationary point (Y*) and its uncertainty were estimated analogously using the gradient vector:

7 $$Var\;\left(Y^\ast\right)=\nabla\;Y^{\ast T}\;\cdot\sum_{}^{}\cdot\;\nabla Y^\ast$$

This dual uncertainty framework enables a quantitative distinction between model-derived stationary points and operationally feasible conditions, supporting the identification of practical process windows for industrial bioprocess design.

To avoid overinterpretation of fitted curvature under limited sample size conditions, model adequacy was evaluated through residual diagnostics, adjusted R², RMSE, and assessment of normality assumptions. Given the OFAT experimental structure and independent univariate modelling strategy, the present framework represents internal model adequacy assessment rather than external predictive validation. The modelling objective was therefore restricted to describing local response behaviour within the tested parameter ranges and quantifying parameter uncertainty, not to developing a globally predictive multivariate response model.

## Results

### Physicochemical characterisation of cheese whey

In the initial phase of bioprocess design, the physicochemical and nutritional profile of CW, intended as the primary substrate, was analysed (Table 1). The results indicate that CW possesses a high carbon load, with a chemical oxygen demand (COD) of approximately 60.0 g/L and a total organic carbon (TOC) content of 45.0 g/L. In addition, total phosphorus (TP) was present at a concentration of approximately 250 mg/L. In contrast, total nitrogen (TN) content of raw CW was measured as 129.9 mg/L.

**Table 1:** Physicochemical characterization of raw CW

Parameters	Unit	Raw CW
COD	g/L	60.0 ± 2.50
TOC	g/L	45.0 ± 1.80
Total Solids	g/L	50.4 ± 1.10
TN	mg/L	129.9 ± 5.4
TP	mg/L	250.0 ± 8.2

Values are expressed as mean ± standard deviation (*n* = 3)

### Screening of LAB strains for SCP production in cheese whey

Seven LAB strains were screened to identify the candidate best adapted to the carbon-rich yet nitrogen-limited characteristics of CW and exhibiting the highest biomass production potential. Analysis of the obtained growth kinetics and biomass yields revealed statistically significant differences in the metabolic capacities of the strains (ANOVA, F(6,14) = 107.16, *p* < 0.001), where the F statistic represents the ratio of between-group variance to within-group variance, indicating that variability among strain means greatly exceeded experimental variability.

Among the tested isolates, *L. garvieae* (0.441 ± 0.024 g/L) and *E faecium* (0.404 ± 0.018 g/L) achieved the highest SCP concentrations and were classified within the statistically highest performance group (Group a) (Table 2). These strains were followed by *L. plantarum and L. lactis subsp. cremoris*, which exhibited intermediate biomass yields, whereas *W. confusa* (0.158 ± 0.006 g/L) showed the lowest biomass formation under the tested conditions.

**Table 2:** Comparative screening of LAB strains for SCP production in CW medium

Strain	SCP Production (g/L)
*L. garvieae*	0.441 ± 0.024^a^
*E. faecium*	0.404 ± 0.018^a^
*L. plantarum*	0.325 ± 0.016^b^
*L.* *lactis cremoris*	0.313 ± 0.018^b^
*L. lactis*	0.263 ± 0.012^c^
*L. sakei*	0.240 ± 0.012^c^
*W. confusa*	0.158 ± 0.006^d^

Values are expressed as mean ± standard deviation (n = 3). Different superscript letters indicate statistically significant differences among strains according to Tukey’s HSD test (p < 0.05)

Although no statistically significant difference was observed between *L. garvieae* and *E. faecium* (*p* > 0.05), *L. garvieae* exhibited the highest mean SCP concentration, which was approximately 9.2% greater than that of *E. faecium*.

### Effect of nitrogen source type on SCP production

To evaluate the effect of nitrogen source type on SCP production, the CW based fermentation medium was supplemented with different plant-derived nitrogen sources at a concentration of 10 g/L. The effects of CE, GLE, and RLE on SCP production by *L. garvieae* were comparatively assessed.

One-way analysis of variance demonstrated that nitrogen source type exerted a highly significant effect on biomass accumulation (ANOVA, F(3,8) = 81.53, *p* < 0.001) (Table 3). Among the tested nitrogen sources, CE supplementation resulted in the highest SCP production, reaching 1.560 ± 0.120 g/L and forming a statistically distinct homogeneous group (Group a; *p* < 0.05).

**Table 3:** Effect of different plant-based nitrogen sources (1% w/v) on SCP production by *L. garvieae*

Nitrogen Source (10 g/L)	SCP Production (g/L)
CE	1.560 ± 0.120^a^
GLE	1.129 ± 0.124^b^
RLE	0.924 ± 0.037^b^
Control	0.441 ± 0.024^c^

Values are expressed as mean ± standard deviation (n = 3). Different superscript letters indicate statistically significant differences among nitrogen sources according to Tukey’s HSD test (p < 0.05)

Both GLE and RLE significantly increased SCP production relative to the control, yielding 1.129 ± 0.124 g/L and 0.924 ± 0.037 g/L, respectively; however, their effects were statistically comparable to each other (Group b; *p* > 0.05). In contrast, the nitrogen-unsupplemented control (CW without added nitrogen source) produced 0.441 ± 0.024 g/L SCP.

### Nutrient enrichment efficiency of chickpea extract

The biochemical composition of chickpea flour and the corresponding chickpea CE obtained after recovery of water-soluble fractions is summarised in Table 4. Raw chickpea flour contained 0.20 ± 0.02 g/g crude protein and 0.033 ± 0.003 g/g total nitrogen. Following extraction, CE exhibited increased protein and nitrogen contents of 0.45 ± 0.01 g/g and 0.070 ± 0.002 g/g, respectively.

**Table 4:** Comparative biochemical composition of chickpea flour and CE

Component (g/g)	Chickpea Flour (Raw Waste)	CE	Enrichment Factor
Crude Protein	0.200 ± 0.020	0.450 ± 0.01	2.25$\:x$
Total Carbohydrate	0.550 ± 0.040	0.420 ± 0.03	0.76$\:x$
Total Nitrogen	0.033 ± 0.003	0.070 ± 0.002	2.12$\:x$
Ash/Minerals	0.030 ± 0.005	0.050 ± 0.01	1.66$\:x$
Crude Lipid	0.070 ± 0.010	ND*	–

Values are expressed as mean ± standard deviation (n = 3). The enrichment factor represents the fold change of each component in CE relative to raw chickpea flour on a dry weight basis. *CE* chickpea extract, *ND* not detected due to separation of the lipid phase during centrifugation (enrichment factor not calculated)

Based on these values, the protein content of CE was enriched by a factor of 2.25 relative to raw chickpea flour, while total nitrogen content increased by a factor of 2.12. Ash content increased from 0.03 ± 0.005 g/g to 0.05 ± 0.01 g/g, corresponding to an enrichment factor of 1.66. Total carbohydrate content decreased from 0.55 ± 0.040 g/g in raw flour to 0.42 ± 0.03 g/g in CE. Crude lipid was not detected in CE following centrifugation.

Values are expressed as mean ± standard deviation (*n* = 3). The enrichment factor represents the fold change of each component in CE relative to raw chickpea flour on a dry weight basis. CE: chickpea extract; ND: not detected due to separation of the lipid phase during centrifugation (enrichment factor not calculated).

### Effect of nitrogen loading on SCP production

**Fig. 1:**
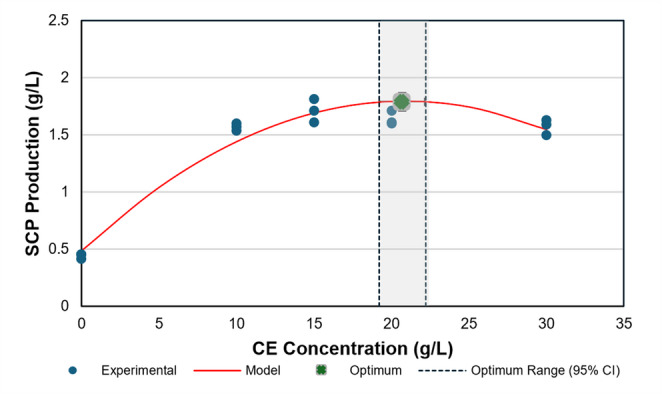
Effect of CE concentration on SCP production with quadratic modelling and model-derived stationary point estimation. Quadratic fit of SCP production as a function of CE concentration. Points represent replicate measurements; the red curve indicates the fitted quadratic model; the green marker denotes the model-based stationary point; dashed vertical lines show the 95% CI for x*

Experimental data describing the response of *L. garvieae* to different CE concentrations (0–30 g/L) were evaluated using one-way analysis of variance and second-order polynomial regression modelling. The tested concentration range (10, 15, 20, and 30 g/L) was selected to encompass nitrogen-limited, literature-equivalent, and potentially saturating supplementation levels. In particular, the intermediate level (15 g/L) approximates the total complex nitrogen content of standard MRS medium (≈ 16 g/L) as reported by Hussein et al. (2015), while lower and higher concentrations were included to assess nitrogen sufficiency and potential saturation behaviour under plant-derived nitrogen supplementation. The concentration-dependent SCP production profile is summarised in Table 5, and the model fit is illustrated in Fig. 1.

**Table 5:** Effect of CE loading rate on SCP production by *L. garvieae*

CE Concentration (g/L)	SCP Production (g/L)
10	1.568 ± 0.033^a^
15	1.714 ± 0.102^a^
20	1.641 ± 0.063^a^
30	1.573 ± 0.064^a^
Control	0.441 ± 0.024^b^

Values are expressed as mean ± standard deviation (n = 3). Different superscript letters indicate statistically significant differences among CE concentrations according to Tukey’s HSD test (p < 0.05)

Analysis of variance revealed that CE concentration exerted a highly significant effect on SCP production (ANOVA, F(4,10) = 212.06, *p* < 0.001). SCP production increased markedly between 0 and 10 g/L CE. At higher concentrations, SCP values ranged from 1.57 to 1.71 g/L, with no statistically significant differences observed among 15 g/L (1.714 ± 0.102 g/L), 20 g/L (1.641 ± 0.063 g/L), and 30 g/L (1.573 ± 0.064 g/L) treatments (Tukey HSD, *p* > 0.05).

A quadratic polynomial provided an adequate empirical description of the relationship between CE concentration and SCP production, showing good agreement with the experimental observations (R² = 0.951; RMSE = 0.118 g/L). The fitted model is given in Eq. 8:

8 $$\:\begin{array}{c}Y_{SCP}=-0.00305x^2+0.12622x+0.48571\end{array}$$

where $\:{Y}_{SCP\:}$is the predicted SCP concentration (g/L) and x is the CE concentration (g/L). Derivative analysis identified a model-based stationary point at approximately 20.7 g/L CE. Using the DM, the stationary point and its uncertainty were estimated as x* = 20.68 ± 0.75 g/L (95% CI: 19.06–22.31 g/L; Fig. 1). The predicted SCP concentration at this point was 1.791 ± 0.041 g/L (95% CI: 1.703–1.879 g/L).

Consistent with the near-flat response across the upper CE range, SCP concentrations between 10 and 30 g/L CE were not statistically distinguishable (*p* > 0.05; Table 5), indicating a broad plateau within the investigated range.

### Effect of carbon source type on SCP production

Using CE fixed at 15 g/L, the effect of different carbon sources (glucose, fructose, lactose, and sucrose) on SCP production by *L. garvieae* was evaluated. SCP yields obtained with each carbon source are summarised in Table 6.

**Table 6:** Effect of different carbon sources on SCP production by *L. garvieae* under selected nitrogen loading conditions

Carbon Source (100 g/L)	SCP Production (g/L)
Sucrose	2.110 ± 0.105^a^
Lactose	1.866 ± 0.048^ab^
Fructose	1.962 ± 0.038^bc^
Glucose	1.739 ± 0.040^c^
Control	1.714 ± 0.102^c^

Values are expressed as mean ± standard deviation (n = 3). Different superscript letters indicate statistically significant differences among carbon sources according to Tukey’s HSD test (p < 0.05)

Analysis of variance demonstrated that carbon source type exerted a statistically significant effect on SCP production (ANOVA, F(4,10) = 14.703, *p* < 0.001). Among the tested substrates, sucrose supplementation (100 g/L) resulted in the highest SCP production, reaching 2.110 ± 0.105 g/L and forming the statistically superior homogeneous group (Group a) (Table 6).

Fructose yielded 1.962 ± 0.040 g/L SCP and was statistically comparable to sucrose (Tukey HSD, *p* = 0.182). Lactose resulted in an intermediate SCP production of 1.866 ± 0.048 g/L and formed a transitional statistical group (Group bc). Glucose supplementation yielded 1.739 ± 0.040 g/L SCP, a value comparable to the control treatment (1.714 ± 0.102 g/L), with both treatments classified within the lowest yield group (Group c).

### Effect of sucrose concentration on SCP production

**Fig. 2:**
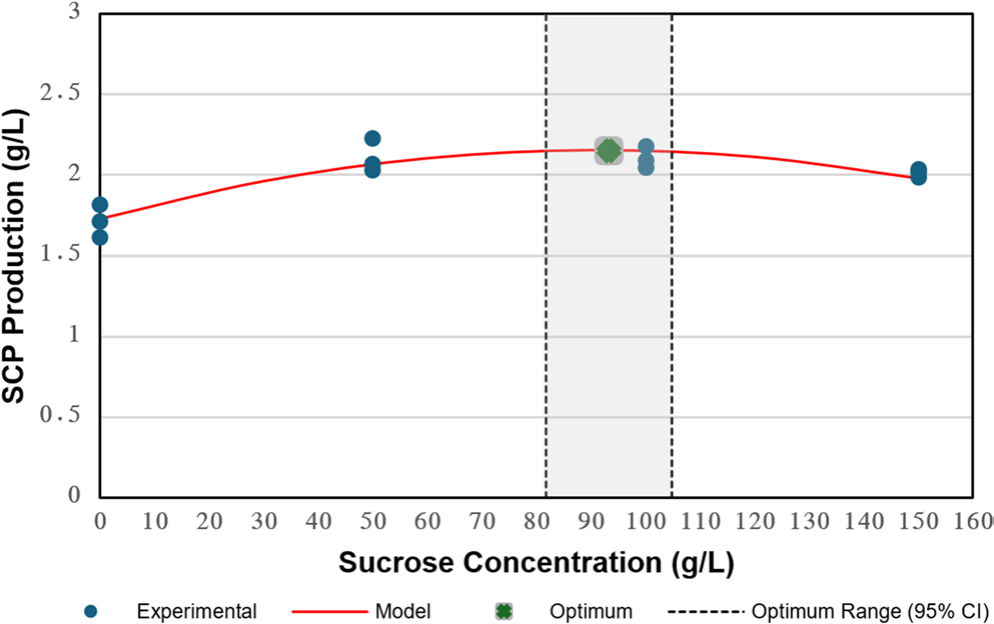
Effect of sucrose concentration on SCP production with quadratic modelling and model-derived stationary point estimation. Points represent replicate measurements; the red curve indicates the fitted model; the green marker denotes the model-based stationary point; dashed vertical lines show the 95% confidence interval for x*

To evaluate the effect of sucrose concentration on SCP production by *L. garvieae*, the dose–response relationship was investigated over a concentration range of 0–150 g/L. Experimental SCP yields obtained at different sucrose concentrations are summarised in Table 7, and the fitted regression model is presented in Fig. 2.

**Table 7:** Dose–response effect of sucrose concentration (g/L) on SCP production by *L. garvieae*

Sucrose Concentration (g/L)	SCP Production (g/L)
50	2.110 ± 0.105^a^
100	2.106 ± 0.066^a^
150	2.009 ± 0.025^a^
Control	1.714 ± 0.102^b^

Values are expressed as mean ± standard deviation (n = 3). Different superscript letters indicate statistically significant differences among sucrose concentrations according to Tukey’s HSD test (p < 0.05)

Analysis of variance indicated that sucrose concentration exerted a statistically significant effect on SCP production (ANOVA, F(3,8) = 15.858, *p* < 0.001). SCP production increased markedly from the control (0 g/L sucrose) to 50 g/L sucrose. However, no statistically significant differences were observed among 50, 100, and 150 g/L treatments (Tukey’s HSD, *p* > 0.05), indicating that biomass production reached a plateau beyond 50 g/L. SCP concentrations at 50, 100, and 150 g/L were 2.110 ± 0.105, 2.106 ± 0.066, and 2.009 ± 0.025 g/L, respectively.

To describe the concentration-dependent production kinetics, experimental data were fitted to a second-order polynomial regression model, which showed acceptable agreement with the observed data (R² = 0.816). The relationship between sucrose concentration and SCP production was expressed by Eq. 9:

The effect of sucrose concentration on SCP production was modelled with a second-order polynomial, which showed acceptable agreement with the experimental observations (R² = 0.816; RMSE = 0.087 g/L). The fitted relationship is given in Eq. 9:

9 $$\:\begin{array}{c}Y_{SCP}=-0.0000490x^2+0.00913x+1.72925\end{array}$$

where $\:{Y}_{SCP}\:$ is the predicted SCP concentration (g/L) and x is the sucrose concentration (g/L). Derivative analysis, combined with the DM, estimated a model-based stationary point at x* = 93.210 ± 5.880 g/L sucrose (95% CI: 79.910–106.510 g/L; Fig. 2). The corresponding predicted SCP concentration at this point was 2.155 ± 0.038 g/L (95% CI: 2.068–2.241 g/L). In line with the experimental contrasts, SCP production measured at 50–150 g/L sucrose did not differ significantly (*p* > 0.050; Table 7), indicating that within the investigated range, increasing sucrose beyond 50 g/L did not yield a statistically supported improvement in SCP concentration.

### Effect of initial pH on SCP production

**Fig. 3:**
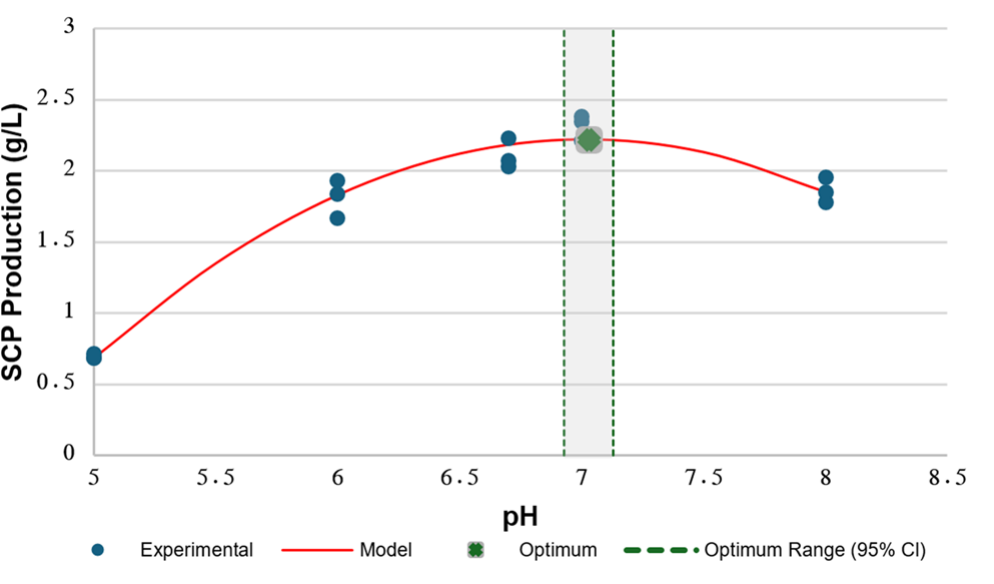
Effect of initial pH on SCP production with quadratic modelling and model-derived stationary point estimation. Quadratic response of SCP production to initial pH. Experimental data, fitted quadratic model, the estimated stationary point, and the 95% CI for pH are shown

The effect of initial pH on SCP production by *L. garvieae* was evaluated over a pH range of 5.0–8.0 under the selected medium composition. In addition to the integer pH levels (5.0, 6.0, 7.0, and 8.0), pH 6.7 was included to provide finer resolution around the near-neutral region, where LAB growth is commonly reported. SCP concentrations obtained at different pH values are summarised in Table 8, and the fitted response curve is presented in Fig. 3.

**Table 8:** Influence of initial pH on SCP production by *L. garvieae*

pH	SCP concentration (g/L)
5.0	0.693 ± 0.018ᵈ
6.0	1.811 ± 0.132ᶜ
6.7	2.110 ± 0.091ᵃᵇ
7.0	2.314 ± 0.090ᵃ
8.0	1.858 ± 0.089ᵇᶜ

Values are expressed as mean ± standard deviation (n = 3). Different superscript letters (a–d) indicate statistically significant differences among pH conditions according to Tukey’s HSD test (p < 0.05)

Analysis of variance demonstrated that initial pH exerted a statistically significant effect on SCP production (ANOVA, F(4,10) = 131.74, *p* < 0.001). SCP production increased markedly as the initial pH approached neutrality, reaching a maximum value of 2.314 ± 0.090 g/L at pH 7.0. According to Tukey’s multiple comparison test, pH 7.0 and pH 6.7 treatments belonged to the same high-yield statistical group (Group a–ab; *p* > 0.05).

At higher and lower pH values, SCP production declined. SCP concentrations of 1.858 ± 0.089 g/L and 1.811 ± 0.132 g/L were obtained at pH 8.0 and pH 6.0, respectively, while the lowest SCP production was observed at pH 5.0 (0.693 ± 0.018 g/L).

To describe the effect of initial pH on SCP production, the experimental data were fitted to a second-order polynomial model, showing strong agreement with the observations (R² = 0.973; RMSE = 0.105 g/L). The fitted relationship is given in Eq. 10:

10 $$\:\begin{array}{c}Y_{SCP}=-0.3718x^2+5.2295x-16.1691\end{array}$$

where $\:{Y}_{SCP}\:$represents the predicted SCP concentration (g/L) and x denotes the initial pH. Derivative analysis identified a model-based stationary point at pH = 7.030 ± 0.050, with a 95% CI of 6.930–7.140 (Fig. 3). The predicted SCP concentration at pH* was 2.220 ± 0.040 g/L.

### Effect of incubation temperature on SCP production

**Fig. 4:**
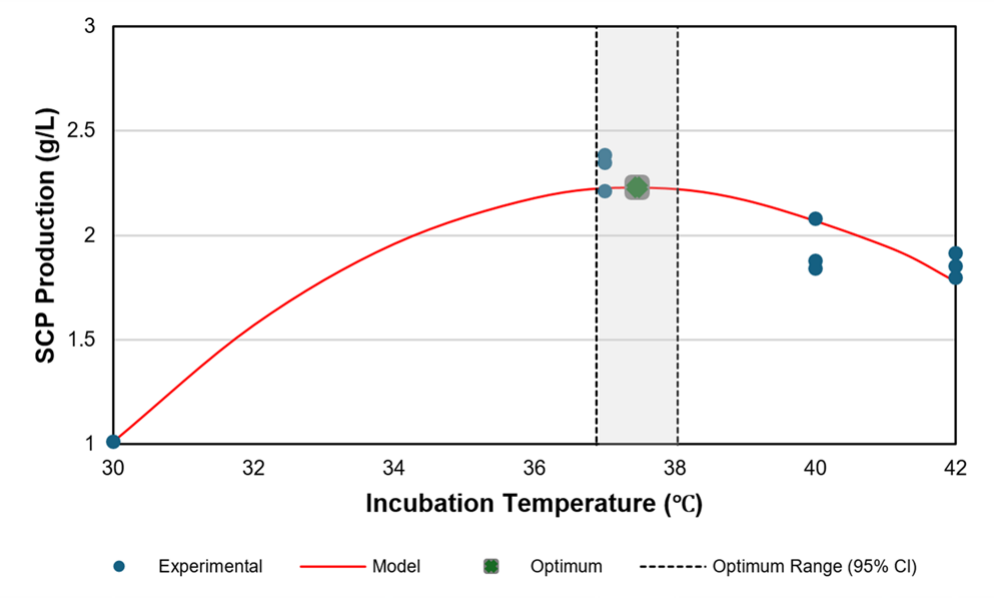
Effect of incubation temperature on SCP production with quadratic modelling and model-derived stationary point estimation. Experimental data, the fitted quadratic model, the estimated stationary point (x*), and its 95% CI are shown

Under the selected medium composition and at the selected initial pH of 7.0, the effect of incubation temperature on SCP production by *L. garvieae* was evaluated over a temperature range of 30–42 °C. SCP concentrations obtained at different temperatures are summarised in Table 9, and the fitted response curve is shown in Fig. 4.

**Table 9:** Effect of incubation temperature on SCP production by *L. garvieae*

Temperature (°C)	SCP concentration (g/L)
30	1.000 ± 0.013ᶜ
37	2.314 ± 0.090ᵃ
40	1.932 ± 0.128ᵇ
42	1.855 ± 0.058ᵇ

Values are expressed as mean ± standard deviation (n = 3). Different superscript letters indicate statistically significant differences among incubation temperatures according to Tukey’s HSD test (p < 0.05)

Analysis of variance demonstrated that incubation temperature exerted a statistically significant effect on SCP production (ANOVA, F(3,8) = 131.35, *p* < 0.001). The highest SCP yield was obtained at 37 °C (2.314 ± 0.090 g/L), which was statistically distinct from all other treatments and formed the highest-performance group (Group a) (Table 9). Increasing the temperature to 40 °C and 42 °C resulted in significantly lower SCP yields of 1.932 ± 0.128 g/L and 1.855 ± 0.058 g/L, respectively, with no statistically significant difference between these two temperatures (Group b; *p* > 0.05). The lowest SCP production was observed at 30 °C (1.000 ± 0.013 g/L), which was significantly lower than all other conditions (Group c).

To describe the effect of incubation temperature on SCP production, the experimental data were fitted to a second-order polynomial model, showing excellent agreement with the observations (R² = 0.939; RMSE = 0.138 g/L). The fitted relationship is given in Eq. 11:

11 $$\:\begin{array}{c}Y_{SCP}=-0.02192x^2+1.64204x-28.5232\end{array}$$

where $\:{Y}_{SCP}$ represents the predicted SCP concentration (g/L) and x denotes the incubation temperature (°C). Derivative analysis identified a model-based stationary point at x* = 37.46 ± 0.30 °C, with a 95% confidence interval of 36.880–38.040 °C (Fig. 4).

### Time-course analysis of SCP production

SCP production by *L. garvieae* was evaluated over an incubation period of 0–72 h. The statistical results and time-dependent production profile are presented in Table 10. One-way ANOVA indicated that incubation time had a highly significant effect on biomass concentration (F(4,10) = 394.04, *p* < 0.001).

**Table 10:** Effect of incubation time on SCP concentration by *L. garvieae*

Incubation Time (h)	SCP Production (g/L)
0 (Control)	0.037 ± 0.005^c^
24	2.314 ± 0.090^b^
36	2.389 ± 0.051^b^
48	2.422 ± 0.101^ab^
72	2.663 ± 0.154^a^

Values are expressed as mean ± standard deviation (n = 3). Different superscript letters indicate statistically significant differences among incubation times according to Tukey’s HSD test (p < 0.05)

As shown in Table 10, the highest SCP concentration was obtained at 72 h (2.663 ± 0.154 g/L), constituting the top statistical group (Group a). The 48 h (2.422 ± 0.101 g/L) and 36 h (2.389 ± 0.051 g/L) time points were statistically similar to each other; however, 48 h showed no significant difference from the 72 h mark (Tukey HSD, $\:p\:=\:0.0648$), placing it in Group ab. The 24 h (2.314 ± 0.090 g/L) and 36 h samples formed Group b, while the 0 h control exhibited the lowest value (Group c).

To describe the time-dependent response within the tested range, a second-order polynomial model was fitted to the experimental data (R² = 0.9365; RMSE = 0.214 g/L). The fitted relationship is expressed in Eq. 12:

12 $$\:\begin{array}{c}Y_{SCP}=-0.000849x^2+0.0944x+0.1558\end{array}$$

where $\:{Y}_{SCP}$ represents the predicted SCP concentration (g/L) and x denotes incubation time (h).

**Fig. 5:**
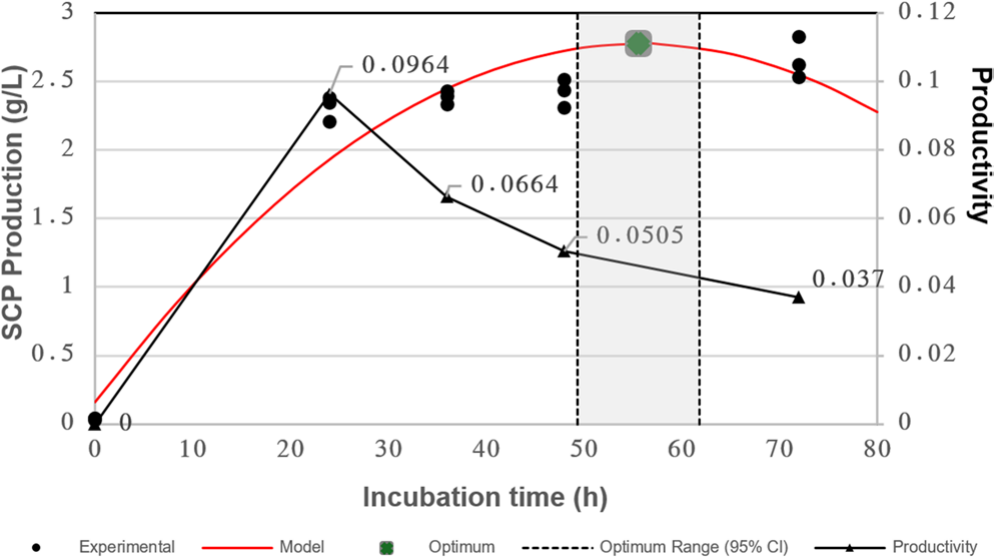
Effect of incubation time on SCP production with quadratic modelling and model-derived stationary point estimation. Experimental data, fitted quadratic model, estimated stationary point (x*), and 95% CI are shown. Volumetric productivity values are also presented

Derivative analysis identified a model-based stationary point at x* = 55.60 ± 3.12 h (95% CI: 49.34–61.86 h). The predicted SCP concentration at x* was 2.780 ± 0.102 g/L (95% CI: 2.576–2.984 g/L) (Fig. 5).

Volumetric productivity $\:{Q}_{p}$ was highest at 24 h (0.096 g/L·h). at 24 h, 0.066 g/L·h at 36 h, 0.050 g/L·h at 48 h, and 0.037 g/L·h at 72 h.

Volumetric productivity (Qₚ) was highest at 24 h (0.096 g/L·h) and subsequently decreased to 0.066 g/L·h at 36 h, 0.050 g/L·h at 48 h, and 0.037 g/L·h at 72 h. While the highest observed biomass concentration occurred at 72 h, approximately 87% of this value was achieved within the first 24 h. Therefore, 24 h was selected as the operational incubation time based on the observed productivity–time trade-off.

### Biochemical characterisation of SCP produced under selected cultivation conditions

The nutritional composition of *L. garvieae* biomass produced under the selected operational conditions (24 h, 37 °C, pH 7.0, 15 g/L CE, 50 g/L sucrose) is a key parameter for evaluating potential application pathways of the product. The biochemical composition of the dried SCP samples is presented in Table 11.

**Table 11:** Biochemical composition of SCP produced under selected operational conditions

Parameters (%)	Mean ± SD
Lipid	2.868 ± 0.337
Nitrogen	9.018 ± 0.441
Protein	56.366 ± 2.754
Carbohydrate	18.004 ± 1.234
Ash	10.554 ± 0.681

According to the analysis results, crude protein was the dominant component of SCP, accounting for 56.366 ± 2.754%. The total nitrogen content was determined to be 9.018 ± 0.441%. Crude lipid content was measured at 2.868 ± 0.337%, while carbohydrate content reached 18.004 ± 1.234%. In addition, the ash content of the SCP biomass was 10.554 ± 0.681% (Table 11).

## Discussion

### Nutrient limitations and strain-specific adaptation in cheese whey

CW is characterised by a pronounced carbon-to-nitrogen (C/N) imbalance, with high organic carbon content but limited assimilable nitrogen. Because cellular protein synthesis is directly constrained by intracellular nitrogen availability, nitrogen limitation imposes a ceiling on biomass accumulation even under carbon-excess conditions. In the untreated CW used in this study (COD ≈ 60 g/L; TN ≈ 130 mg/L), *L. garvieae* produced 0.441 ± 0.024 g/L SCP, confirming that carbon abundance alone was insufficient to sustain further biomass synthesis.

Strain screening revealed marked interspecies variability in adaptation to this nutritionally imbalanced matrix (Table 2). Among the seven LAB strains evaluated, *L. garvieae* achieved the highest biomass concentration under untreated conditions, followed by *E. faecium*. This pattern suggests differential capacity for nitrogen assimilation, intracellular recycling, or carbon allocation efficiency under C/N imbalance. In contrast, the lower SCP yields observed for *L. plantarum*,* L. lactis subsp. cremoris*,* L. sakei*, and *W. confusa* indicate strain-specific metabolic constraints limiting biomass formation in nitrogen-deficient whey.

Dedicated studies on SCP-oriented biomass production by *L. garvieae* remain limited. However, genomic and physiological data indicate broad environmental adaptability, including tolerance to wide pH (4.5–9.6) and temperature (10–45 °C) ranges (Aguado-Urda et al. 2013). This physiological plasticity may partly explain its comparatively robust performance in the present whey system. The response of *E. faecium* is consistent with previous reports demonstrating effective whey-based biomass production following process optimisation (Guerra et al. 2010), supporting the view that selected LAB taxa can adapt efficiently to dairy-derived substrates.

Beyond strain-level differences, literature evidence indicates that substantially higher biomass titres can be achieved in whey systems when complex nitrogen sources and defined mineral inputs are provided. Mondragón-Parada et al. (2006) obtained ~ 2.0 g/L LAB biomass using ammonium salts and yeast extract, while Nanjaiah et al. (2024) reported 5.56 ± 0.34 g/L dry cell weight under response surface–optimised conditions with yeast extract and mineral supplementation. These studies reflect performance under nutrient-enriched strategies. In contrast, the present work evaluates strain behaviour within a valorisation-oriented framework employing a waste-derived nitrogen supplement (CE) rather than commercial formulations, which fundamentally alters process objectives and nutrient economics.

While *L. garvieae* has been reported as a pathogen in specific aquaculture contexts, pathogenicity is strain-specific and associated with defined virulence determinants rather than representing a uniform species-level characteristic. The isolate used in this study originated from a dairy matrix (Onur and Önlü 2021), and the species has been reported in raw milk and cheeses (Abdelfatah and Mahboub 2018). Given documented intra-species genomic variability (Aguado-Urda et al. 2013), safety evaluation should therefore be considered at the strain level rather than inferred solely from taxonomic designation.

Beyond taxonomic considerations, it is also important to position these findings within the broader LAB fermentation literature. Industrial LAB systems are predominantly designed for metabolite production—such as lactic acid, exopolysaccharides, bacteriocins, vitamins, and flavour compounds—rather than for maximising cell mass (Li et al., 2025; Wang et al., 2021; Yang et al., 2025; Zeidan et al., 2017). In such metabolite-centred processes, microbial biomass is typically regarded as an intermediate requirement for product synthesis and often treated as residual material rather than as the principal output. In contrast, the present study repositions LAB biomass as the principal product stream within a whey-based system, thereby reorienting fermentative carbon allocation from metabolite-centred valorisation toward biomass-focused protein production under waste-derived nitrogen supplementation.

### Nitrogen source enrichment and SCP yield response

The substantial increase in SCP production following CE supplementation indicates a more favourable biomass response compared with lentil-derived extracts under the tested conditions. Chickpea proteins are reported to provide a balanced amino acid composition and may support improved nitrogen assimilation by LAB compared with lentil substrates, thereby enhancing biomass formation under nitrogen-limited conditions (Begum et al. 2023; Boye et al. 2010; Khazaei et al. 2019; Kröper et al. 2025). In addition to protein-derived nitrogen, CE contains soluble carbohydrates such as galacto-oligosaccharides and low-molecular-weight sugars that can be rapidly metabolised by LAB and may exert synergistic effects on growth (Martins et al. 2023). Previous studies have reported enhanced LAB biomass formation in chickpea-supplemented fermentations (Amadei et al. 2025; Kahve and Bayrak 2025; Sáez et al. 2018; Zhang et al. 2022), further supporting the suitability of CE as an effective nitrogen enrichment strategy.

The water-based extraction applied in this study effectively concentrated the nutritionally relevant fractions of chickpea flour. The resulting extract exhibited higher nitrogen content (1.83% vs. 1.28% in whole flour) while reducing carbohydrate (44.90% vs. 58.40%) and lipid (0.17% vs. 4.94%) fractions. This compositional shift indicates selective enrichment of the protein-rich fraction and removal of components that do not directly contribute to microbial biomass formation. Increasing assimilable nitrogen within a reduced volumetric input is particularly relevant for SCP-oriented fermentations, where nitrogen supply directly governs cell growth.

Despite the positive response to CE supplementation, the quadratic model predicted a stationary point at x* = 20.680 g/L CE (95% CI: 19.060–22.310 g/L). However, experimental data showed that SCP titres between 10 and 30 g/L CE were not statistically distinguishable (*p* > 0.050; Table 5; Fig. 1), indicating that biomass production approached a practical plateau within the tested range. This plateau-like behaviour reinforces that the response maximum identified by the quadratic model does not correspond to a biologically distinct productivity regime within the tested domain. The negative curvature term (b₂ = −3.050 × 10⁻³) supports a saturation-type response, with diminishing incremental gains at higher CE concentrations. This behaviour suggests that factors other than nitrogen availability became limiting beyond mid-range supplementation. Potential secondary constraints include carbon source depletion, metabolite accumulation (e.g., LA), pH drift, or trace element limitation, all of which are known to restrict LAB growth under nitrogen-replete conditions (Hofvendahl and Hahn–Hägerdal 2000). At elevated extract concentrations, osmotic effects or matrix-associated compounds may also contribute to non-linear growth responses (Alajaji and El-Adawy 2006). Importantly, the absence of statistically significant differences across the 10–30 g/L CE range indicates a saturation trend rather than definitive inhibition.

From a process design perspective, distinguishing between a model-derived stationary point and a practical operational setpoint is essential. Increasing CE from 15 to 20.680 g/L represents approximately a 38% increase in substrate input, while the model-implied biomass gain is modest (Δŷ ≈ 0.099 g/L) and not experimentally supported as a significant improvement. The calculated marginal benefit (Δŷ/Δx ≈ 0.017 g SCP per g CE) indicates that additional nitrogen input yielded minimal biomass return within this range, suggesting that nitrogen was unlikely to remain the primary growth-limiting factor within this concentration range. Accordingly, 15 g/L CE was selected as the operational concentration, balancing yield stability with substrate economy.

This explicit distinction between model-derived stationary points and practically selected operating levels addresses a recurrent limitation in fermentation studies, where stationary points are often adopted without evaluation of marginal benefit. Previous whey-based SCP investigations have largely prioritised maximum biomass concentration or protein content (Guerra et al. 2010; Somaye et al. 2008) without systematically assessing incremental substrate efficiency. Integrating marginal benefit analysis at the experimental stage strengthens process interpretation by linking laboratory-scale observations to techno-economic and sustainability considerations.

Importantly, the chickpea flour used in this study was sourced from expired batches unsuitable for human consumption, representing a distinct waste valorisation pathway. Although chickpea-based substrates have been used in sourdough fermentation and nutritional fortification contexts (Grasso et al. 2022; Xing et al. 2020), these applications rely on food-grade materials. In contrast, converting expired legume flour into a nitrogen-rich aqueous extract for microbial protein production represents a dual-waste valorisation strategy integrating dairy whey and substandard legume flour within a single bioprocess.

This approach differentiates the present study from conventional whey bioconversion systems relying on commercial yeast extract or inorganic nitrogen salts (Amini et al. 2025; Guerra et al. 2010). The integration of a second waste-derived nitrogen source as the primary enrichment strategy within a whey-based SCP framework remains limited in the current literature. The solvent-free, water-based extraction method employed here avoids organic solvents, alkaline hydrolysis, and enzymatic treatments, thereby maintaining low operational complexity. From a circular bioeconomy perspective, coupling expired legume flour valorisation with dairy whey conversion simultaneously addresses two food-industry waste streams, potentially improving environmental performance and resource efficiency relative to single-waste or virgin-input strategies.

### Carbon source selection and metabolic constraints

The superior SCP production observed with sucrose supplementation is consistent with its favourable metabolic compatibility with *L. garvieae*. In *Lactococcus* species, sucrose utilisation is mediated by dedicated scr/sac operons linked to the phosphoenolpyruvate-dependent phosphotransferase system, enabling efficient intracellular conversion of sucrose into metabolically available hexoses (Luesink et al. 1999; Rauch and de Vos 1992). Comparable sucrose-associated genetic determinants have also been described within the Lactococcus genus, including *L. garvieae*. Consistent with this metabolic framework, genomic analysis of the strain used in this study confirmed the presence of the sucrose-6-phosphate hydrolase gene, supporting its capacity for intracellular sucrose metabolism (NCBI, 2021).

By contrast, the lower SCP yield observed with glucose supplementation may be related to carbon catabolite repression and osmotic effects. Glucose is recognised as a strong catabolite repressor in LAB, which can constrain biosynthetically favourable routes, and its high molar contribution at equivalent mass concentrations may further increase osmotic burden (Gu et al. 2024). Together, these mechanisms provide a plausible explanation for the observed differences among carbon sources.

Although fructose produced SCP levels statistically comparable to sucrose, sucrose was retained as the operational carbon source for subsequent process evaluation due to its broader industrial availability and cost advantages, particularly when supplied as molasses.

Regarding sucrose dose–response, the limited difference between the model-based stationary point (x* ≈ 93.210 g/L) and the experimentally observed response at 50 g/L indicates that SCP production approached a saturation-like regime well below the model-derived point estimate (Table 7; Fig. 2). In the tested domain (50–150 g/L), increasing sucrose concentration did not yield a statistically supported improvement in SCP titre (*p* > 0.050), indicating diminishing marginal returns and a plateau-like response within the tested domain. Although the quadratic model identifies a stationary point, the absence of statistically supported differences across 50–150 g/L suggests that this point does not correspond to a practically distinct productivity regime under the investigated conditions.

Substrate inhibition was evaluated operationally using final SCP concentration as the response variable. Within 50–150 g/L, no statistically significant decrease in SCP titre was detected (*p* > 0.050; Table 7), indicating that clear inhibitory effects were not supported within the investigated range. Nevertheless, the fitted negative curvature (β₂ = −4.900 × 10⁻⁵) and the slightly lower mean at 150 g/L suggest a trend toward reduced process efficiency at elevated sucrose concentrations, plausibly associated with increased medium osmolality and shifts in metabolic allocation reported for LAB (González-Garcinuño et al. 2017; Molina-Höppner et al. 2004; van der Meulen et al. 2017). Additional response indicators (e.g., residual sugar, acid accumulation, viability, or productivity) would be required to characterise potential inhibitory effects beyond titre-based evaluation.

From an applied standpoint, increasing sucrose above 50 g/L increases carbon input substantially while delivering no statistically supported improvement in SCP titre within the tested range. Therefore, 50 g/L sucrose was selected as the operational concentration for subsequent steps.

### Process condition responses and physiological constraints

The integrated methodological framework combined OFAT screening with quadratic polynomial fitting and DM–based confidence intervals to describe local univariate response behaviour in the whey-based SCP system. While RSM/DOE designs can capture interaction effects, the present analysis was restricted to single-factor responses within the tested ranges, and uncertainty in model-derived stationary points was quantified via analytical variance propagation. DM enables direct estimation of confidence intervals without simulation-based resampling (e.g., bootstrap or Monte Carlo) and has been used in bioprocess applications (Bhonsale et al., 2021; Petersen and Jørgensen 2014). Within this OFAT structure, quadratic polynomials were used as empirical descriptors of curvature rather than full second-order response surfaces including interaction terms (Khuri and Mukhopadhyay 2010; Mandenius and Brundin 2008; Montgomery, 2017).

The close agreement between the experimentally observed highest SCP value at pH 7.0 and the model-derived stationary point (pH* = 7.030; 95% CI: 6.930–7.140) indicates that near-neutral conditions yielded the highest SCP formation within the tested pH range (Table 8; Fig. 3). This behaviour is consistent with the reported physiological adaptability of *L. garvieae* across diverse habitats and pH conditions (Aguado-Urda et al. 2013; Icer et al. 2023). The pronounced reduction in SCP production under acidic conditions, and the moderate decline at alkaline pH, align with established physiological constraints in LAB. Acidic environments may induce cytoplasmic acidification and disrupt proton motive force–dependent transport, whereas alkaline conditions can impair membrane potential and enzyme activity, collectively reducing nutrient uptake efficiency and growth-associated biomass formation (Patel et al. 2020; Rubiã and Mesquita 2018).

Similarly, the experimentally observed highest SCP value at 37 °C closely matched the model-derived stationary point (37.460 °C; 95% CI: 36.880–38.040 °C), consistent with mesophilic growth characteristics under the studied conditions (Table 9; Fig. 4). Reduced SCP titres at elevated temperatures are consistent with thermal stress–related impairment of cellular processes, whereas lower temperatures constrain enzymatic activity and membrane fluidity, limiting nutrient transport and biomass accumulation (Aguado-Urda et al. 2013; Alighialo et al. 2019; Davey 1991).

From a process perspective, evaluation based solely on final biomass concentration may not adequately reflect batch performance. At 24 h, SCP reached 2.314 g/L, corresponding to approximately 87% of the highest observed concentration at 72 h (2.663 g/L), indicating that most biomass formation occurred during the initial cultivation phase.

Volumetric productivity analysis reinforces this interpretation. The highest Qₚ value (Eq. 13) was obtained at 24 h (0.096 g/L·h), while continued incubation resulted in a decline to 0.037 g/L·h at 72 h, demonstrating diminishing time-normalised returns despite continued increases in absolute biomass.

13 $$\:\begin{array}{c}Q_p=\frac{P_{final}-P_{initial}}t\end{array}$$

where P denotes biomass concentration (g/L) and t represents fermentation time (h).

The rapid biomass accumulation observed within the first 24 h aligns with reports describing high early growth kinetics of L. garvieae, which can approach near-maximum cell densities within the first operational day under favourable cultivation conditions. In several fermentation matrices, cell density has been reported to show limited additional increase after approximately 48 h under nutrient-constrained conditions. The reduction in volumetric productivity during extended incubation is consistent with progression toward late exponential or early stationary phase. At this stage, cumulative environmental stressors such as nutrient depletion and metabolite accumulation may compromise membrane integrity and alter permeability, which can lead to intracellular leakage and reduced effective biomass formation rates (Kozakai et al., 2020; Soemarie et al., 2025). Although net biomass accumulation continued up to 72 h, the magnitude of increase was comparatively limited and time-normalised productivity declined. Consequently, the highest time-normalised returns were achieved during the early growth phase, supporting the selection of 24 h as the operational incubation time prior to the onset of growth-limiting physiological constraints.

Within this framework, the quadratic time model represents a statistical description of local response curvature rather than a mechanistic growth equation. The model-derived stationary point (≈ 55.60 h; R² = 0.92–0.94) indicates where the fitted marginal increase approaches zero within the polynomial structure; however, operational selection was based on the observed productivity–titre trade-off, for which 24 h provided the highest time-normalised return within the tested window.

Based on combined consideration of total biomass and volumetric productivity, 24 h was selected as the operational incubation time, reflecting a trade-off between biomass yield and residence time. The consistent application of quadratic modelling across substrate concentration, pH, temperature, and time maintains methodological coherence within the OFAT framework and allows estimation of response curvature and associated confidence intervals under a unified statistical structure.

### Biochemical profile and biorefinery relevance of the produced SCP

The crude protein content of the produced SCP (56.37%) falls within the FAO reference range for bacterial SCP (50–83%) (FAO, 2025) and is consistent with nitrogen assimilation into intracellular protein under the applied cultivation conditions. The measured nitrogen content (9.01%) is likewise within values commonly reported for bacterial SCP.

Direct comparison of protein fractions across studies should be interpreted cautiously, as biomass composition is strongly influenced by nitrogen availability, medium formulation, mineral balance, growth phase, and analytical nitrogen-to-protein conversion factors. Reported protein fractions therefore reflect cultivation regime and nutrient context rather than inherent species-level protein potential.

The low crude lipid content (2.87%), relative to typical bacterial biomass values (8–10%), may be advantageous in applications where lower lipid content is preferred (Demirel and Demirel 2018). In contrast, the ash content (10.55%) was located at the upper end of reported ranges, indicating incorporation of minerals originating from CW and CE into the biomass and suggesting potential value as a mineral-enriched component.

The carbohydrate fraction (18%) is consistent with literature values for bacterial SCP (FAO, 2025) and is primarily attributable to cell wall–associated polysaccharides. Overall, the compositional profile characterises the produced SCP as a protein-rich, low-lipid, and mineral-containing biomass.

Considering the high chemical oxygen demand, nitrogen, and phosphorus load of CW, its conversion into a microbial biomass containing approximately 56% protein represents a potential value-recovery pathway within a circular bioeconomy context. Within this framework, the produced SCP may serve as a protein-rich intermediate for further bioprocessing applications, subject to strain-level safety evaluation where required.

Taken together, the present study extends whey-based SCP research in three key directions. First, nitrogen enrichment was achieved using a second waste stream derived from expired chickpea flour, establishing a dual-waste valorisation framework rather than relying on commercial nitrogen sources. Second, model-derived stationary points were explicitly distinguished from operational setpoints through marginal benefit evaluation, separating statistical curvature from practically meaningful productivity regimes. Third, incubation time was selected based on the productivity–titre trade-off rather than maximum final biomass alone. This combined approach repositions LAB biomass as the primary product in a whey system and introduces an uncertainty-informed process evaluation framework that remains limited in current SCP literature.

## Conclusion

This study developed a whey-based SCP production framework using *L. garvieae* within a dual waste-valorisation configuration integrating CW and expired CE. Sequential OFAT evaluation combined with second-order polynomial modelling and DM–based uncertainty analysis enabled quantitative differentiation between model-derived stationary points and operationally selected process conditions. Nitrogen supplementation with chickpea extract substantially increased biomass formation relative to untreated whey, while sucrose was identified as the most effective co-substrate. Although quadratic modelling identified stationary points for nitrogen loading, sucrose concentration, pH, temperature, and time, operational conditions were selected based on statistical contrasts and marginal productivity rather than solely on curvature-derived maxima. Incubation at 24 h was chosen as the operational time due to the highest observed volumetric productivity (0.096 g/L·h), despite a higher biomass concentration at 72 h. The produced biomass contained approximately 56% protein (dry weight), within the FAO reference range for bacterial SCP, demonstrating effective nitrogen incorporation under waste-derived supplementation. The use of expired legume flour as a nitrogen source establishes a dual-waste valorisation strategy distinct from conventional whey systems relying on commercial yeast extract or inorganic nitrogen salts. Conceptually, the study contributes an uncertainty-aware modelling framework within an OFAT structure, explicitly distinguishing model-derived stationary points from practically justified operating conditions. While the approach does not replace multivariate RSM-based optimisation, it provides statistically supported local response characterisation under laboratory-scale constraints. Future work should address process intensification, scale-up validation, and strain-level safety assessment prior to downstream application requiring regulatory evaluation.

## Supplementary Information

Below is the link to the electronic supplementary material.

Supplementary Material 1 (PDF 119 KB)

## Data Availability

No datasets were generated or analysed during the current study.
