# Supplementary material for: Valorisation of cheese whey into single-cell protein by Lactococcus garvieae: dual waste nitrogen supplementation and uncertainty-informed process evaluation
Source: World J Microbiol Biotechnol. 2026 Apr 21;42(5):227. doi: 10.1007/s11274-026-04911-3 (PMC13095961; doi:10.1007/s11274-026-04911-3)
Supplement: Supplementary file 1 — Supplementary Material 1 (PDF 119 KB) [file 11274_2026_4911_MOESM1_ESM.pdf]

Supplementary Material

Valorisation of cheese whey into single-cell protein by Lactococcus garvieae: dual waste nitrogen supplementation and uncertainty-informed process evaluation

Table S1. Quadratic regression model parameters and validation metrics

All models follow the general form $Y = \beta_0 + \beta_1 x + \beta_2 x^2$. Parameters were estimated by ordinary least squares (OLS) using individual replicate observations. Residual normality was assessed by the Shapiro–Wilk (S-W) test at $\alpha = 0.05$.

Model	β_0 ± SE	β_1 ± SE	β_2 ± SE	R^2	R^2adj	RMSE	S-W p
CE (g/L)	0.486 ± 0.066	0.1262 ± 0.0094	-3.05×10^{-3} ± 2.98×10^{-4}	0.951	0.938	0.118	0.380
Sucrose (g/L)	1.729 ± 0.049	9.13×10^{-3} ± 1.56×10^{-3}	-4.90×10^{-5} ± 1.00×10^{-5}	0.816	0.747	0.087	0.815
pH	−16.169 ± 1.107	5.230 ± 0.347	−0.372 ± 0.027	0.973	0.965	0.105	0.709
Temp. (°C)	−28.523 ± 3.454	1.642 ± 0.196	−0.0219 ± 0.0027	0.939	0.917	0.138	0.099
Time (h)	0.156 ± 0.154	0.0944 ± 0.0091	-8.49×10^{-4} ± 1.20×10^{-4}	0.937	0.919	0.275	0.322

Table S2. Delta Method optimum estimates with 95% confidence intervals

Theoretical optima (x^*) were derived analytically as $x^* = -\beta_1/(2\beta_2)$. Uncertainty was propagated using the Delta Method based on the variance–covariance matrix of the regression coefficients. Confidence intervals were constructed using the t-distribution with $v = n - 3$ degrees of freedom.

Parameter	x^* ± SE	95% CI (x^*)	Y^* ± SE (g/L)	95% CI (Y^*)	n
CE (g/L)	20.68 ± 0.75	[19.06, 22.31]	1.791 ± 0.041	[1.703, 1.879]	15
Sucrose (g/L)	93.21 ± 5.88	[79.91, 106.51]	2.155 ± 0.038	[2.068, 2.241]	12
pH	7.03 ± 0.05	[6.92, 7.15]	2.222 ± 0.036	[2.145, 2.300]	15
Temp. (°C)	37.46 ± 0.30	[36.79, 38.12]	2.230 ± 0.068	[2.076, 2.383]	12
Time (h)	55.60 ± 3.27	[48.47, 62.74]	2.780 ± 0.094	[2.575, 2.986]	15

Note: The time model optimum (55.6 h) represents a mathematical artifact of the quadratic function. Biomass concentration is a cumulative parameter that does not decline under the conditions tested; the predicted post-optimum decrease is inherent to the symmetric parabolic form and does not reflect biological reality.

Table S3. Modified Gompertz model parameters for fermentation time-course

The Modified Gompertz model $Y(t) = A \cdot \exp\{-\exp[(\mu_{max} \cdot e / A)(\lambda - t) + 1]\}$ was fitted to the time-course SCP data as a mechanistically appropriate alternative to the quadratic model. Unlike the quadratic function, the Gompertz model captures the asymptotic plateau behaviour characteristic of cumulative biomass production.

Parameter	Symbol	Estimate ± SE	Unit	95% CI
Maximum asymptotic SCP	A	2.514 ± 0.049	g/L	[2.408, 2.620]
Maximum specific rate	μ_{max}	0.144 ± 0.021	g/L·h	[0.098, 0.190]
Lag phase duration	λ	2.43 ± 2.31	h	[−2.61, 7.47]

Table S4. Model comparison: Quadratic vs Modified Gompertz for fermentation time

Metric	Quadratic	Modified Gompertz	Preferred
R^2	0.937	0.986	**Gompertz**
R^2adj	0.919	0.982	**Gompertz**
RMSE	0.275	0.131	**Gompertz**
AIC	−36.1	−58.4	**Gompertz**
BIC	−34.0	−56.3	**Gompertz**
Shapiro–Wilk p	0.322	0.200	**Both pass**
Biological validity	Predicts decline	Predicts plateau	**Gompertz**

Table S5. Modified Gompertz model predictions (0–72 h)

Predicted SCP concentrations at 6 h intervals. Amax = 2.514 g/L.

Time (h)	Predicted SCP (g/L)	% of Amax	Time (h)	Predicted SCP (g/L)	% of Amax	Time (h)	Predicted SCP (g/L)	% of Amax
0.00	0.0475	1.9	24.48	2.3033	91.6	48.60	2.5089	99.8
0.36	0.0589	2.3	24.84	2.3143	92.1	48.96	2.5092	99.8
0.72	0.0723	2.9	25.20	2.3248	92.5	49.32	2.5094	99.8
1.08	0.0878	3.5	25.56	2.3347	92.9	49.68	2.5097	99.8
1.44	0.1054	4.2	25.92	2.3442	93.2	50.04	2.5099	99.8
1.80	0.1253	5.0	26.28	2.3531	93.6	50.40	2.5101	99.8
2.16	0.1476	5.9	26.64	2.3616	93.9	50.76	2.5103	99.9
2.52	0.1723	6.9	27.00	2.3697	94.3	51.12	2.5105	99.9
2.88	0.1994	7.9	27.36	2.3773	94.6	51.48	2.5107	99.9
3.24	0.2290	9.1	27.72	2.3846	94.9	51.84	2.5109	99.9
3.60	0.2609	10.4	28.08	2.3915	95.1	52.20	2.5111	99.9
3.96	0.2953	11.7	28.44	2.3980	95.4	52.56	2.5112	99.9
4.32	0.3319	13.2	28.80	2.4042	95.6	52.92	2.5114	99.9
4.68	0.3706	14.7	29.16	2.4101	95.9	53.28	2.5115	99.9
5.04	0.4114	16.4	29.52	2.4156	96.1	53.64	2.5117	99.9
5.40	0.4541	18.1	29.88	2.4209	96.3	54.00	2.5118	99.9
5.76	0.4985	19.8	30.24	2.4259	96.5	54.36	2.5119	99.9
6.12	0.5445	21.7	30.60	2.4306	96.7	54.72	2.5120	99.9
6.48	0.5919	23.5	30.96	2.4351	96.9	55.08	2.5121	99.9
6.84	0.6405	25.5	31.32	2.4393	97.0	55.44	2.5122	99.9
7.20	0.6901	27.5	31.68	2.4433	97.2	55.80	2.5123	99.9

7.56	0.7405	29.5	32.04	2.4471	97.3	56.16	2.5124	99.9
7.92	0.7916	31.5	32.40	2.4507	97.5	56.52	2.5125	99.9
8.28	0.8431	33.5	32.76	2.4541	97.6	56.88	2.5126	99.9
8.64	0.8949	35.6	33.12	2.4574	97.7	57.24	2.5127	99.9
9.00	0.9467	37.7	33.48	2.4604	97.9	57.60	2.5127	99.9
9.36	0.9985	39.7	33.84	2.4633	98.0	57.96	2.5128	100.0
9.72	1.0501	41.8	34.20	2.4661	98.1	58.32	2.5129	100.0
10.08	1.1013	43.8	34.56	2.4687	98.2	58.68	2.5129	100.0
10.44	1.1520	45.8	34.92	2.4711	98.3	59.04	2.5130	100.0
10.80	1.2021	47.8	35.28	2.4734	98.4	59.40	2.5130	100.0
11.16	1.2515	49.8	35.64	2.4756	98.5	59.76	2.5131	100.0
11.52	1.3000	51.7	36.00	2.4777	98.6	60.12	2.5131	100.0
11.88	1.3476	53.6	36.36	2.4797	98.6	60.48	2.5132	100.0
12.24	1.3943	55.5	36.72	2.4815	98.7	60.84	2.5132	100.0
12.60	1.4398	57.3	37.08	2.4833	98.8	61.20	2.5133	100.0
12.96	1.4843	59.0	37.44	2.4850	98.8	61.56	2.5133	100.0
13.32	1.5276	60.8	37.80	2.4865	98.9	61.92	2.5134	100.0
13.68	1.5696	62.4	38.16	2.4880	99.0	62.28	2.5134	100.0
14.04	1.6105	64.1	38.52	2.4894	99.0	62.64	2.5134	100.0
14.40	1.6501	65.6	38.88	2.4908	99.1	63.00	2.5135	100.0
14.76	1.6884	67.2	39.24	2.4920	99.1	63.36	2.5135	100.0
15.12	1.7255	68.6	39.60	2.4932	99.2	63.72	2.5135	100.0
15.48	1.7613	70.1	39.96	2.4943	99.2	64.08	2.5135	100.0
15.84	1.7958	71.4	40.32	2.4954	99.3	64.44	2.5136	100.0
16.20	1.8291	72.8	40.68	2.4964	99.3	64.80	2.5136	100.0
16.56	1.8611	74.0	41.04	2.4974	99.3	65.16	2.5136	100.0
16.92	1.8919	75.3	41.40	2.4983	99.4	65.52	2.5136	100.0
17.28	1.9214	76.4	41.76	2.4991	99.4	65.88	2.5137	100.0
17.64	1.9498	77.6	42.12	2.4999	99.4	66.24	2.5137	100.0
18.00	1.9770	78.6	42.48	2.5007	99.5	66.60	2.5137	100.0
18.36	2.0031	79.7	42.84	2.5014	99.5	66.96	2.5137	100.0
18.72	2.0281	80.7	43.20	2.5021	99.5	67.32	2.5137	100.0
19.08	2.0520	81.6	43.56	2.5028	99.6	67.68	2.5137	100.0
19.44	2.0748	82.5	43.92	2.5034	99.6	68.04	2.5138	100.0
19.80	2.0967	83.4	44.28	2.5040	99.6	68.40	2.5138	100.0
20.16	2.1175	84.2	44.64	2.5045	99.6	68.76	2.5138	100.0
20.52	2.1375	85.0	45.00	2.5050	99.6	69.12	2.5138	100.0
20.88	2.1565	85.8	45.36	2.5055	99.7	69.48	2.5138	100.0
21.24	2.1746	86.5	45.72	2.5060	99.7	69.84	2.5138	100.0
21.60	2.1919	87.2	46.08	2.5064	99.7	70.20	2.5138	100.0
21.96	2.2083	87.8	46.44	2.5068	99.7	70.56	2.5138	100.0
22.32	2.2240	88.5	46.80	2.5072	99.7	70.92	2.5138	100.0
22.68	2.2389	89.1	47.16	2.5076	99.7	71.28	2.5139	100.0
23.04	2.2531	89.6	47.52	2.5079	99.8	71.64	2.5139	100.0
23.40	2.2666	90.2	47.88	2.5083	99.8	72.00	2.5139	100.0
23.76	2.2795	90.7	48.24	2.5086	99.8			
24.12	2.2917	91.2						

Table S6. Derived kinetic parameters from the Modified Gompertz model

Derived Parameter	Value	Unit
Doubling time (td)	4.45	h
Time to 50% of Amax	13.0	h
Time to 90% of Amax	23.3	h
Time to 95% of Amax	27.9	h
Time to 99% of Amax	38.5	h
Maximum instantaneous rate (at inflection)	0.144	g/L·h
Inflection point time	11.6	h
SCP at inflection point	0.925	g/L

Table S7. Variance–covariance matrices of model coefficients

These matrices were used for Delta Method uncertainty propagation (Eq. 5–7 in the main text). Diagonal elements represent parameter variances; off-diagonal elements represent covariances.

CE concentration ($Y = \beta_0 + \beta_1 x + \beta_2 x^2$)			
	β_0	β_1	β_2
β_0	6.554×10^{-3}	-7.015×10^{-4}	1.647×10^{-5}
β_1	-7.015×10^{-4}	1.324×10^{-4}	-3.952×10^{-6}
β_2	1.647×10^{-5}	-3.952×10^{-6}	1.317×10^{-7}
Sucrose concentration ($Y = \beta_0 + \beta_1 x + \beta_2 x^2$)			
	β_0	β_1	β_2
β_0	5.072×10^{-4}	-1.121×10^{-5}	5.339×10^{-8}
β_1	-1.121×10^{-5}	5.232×10^{-7}	-3.203×10^{-9}
β_2	5.339×10^{-8}	-3.203×10^{-9}	2.136×10^{-11}
pH ($Y = \beta_0 + \beta_1 x + \beta_2 x^2$)			
	β_0	β_1	β_2
β_0	3.923×10^{-1}	-1.224×10^{-1}	9.322×10^{-3}
β_1	-1.224×10^{-1}	3.844×10^{-2}	-2.948×10^{-3}
β_2	9.322×10^{-3}	-2.948×10^{-3}	2.274×10^{-4}
Temperature ($Y = \beta_0 + \beta_1 x + \beta_2 x^2$)			
	β_0	β_1	β_2
β_0	1.193×10^{1}	-6.768×10^{-1}	9.433×10^{-3}
β_1	-6.768×10^{-1}	3.850×10^{-2}	-5.379×10^{-4}
β_2	9.433×10^{-3}	-5.379×10^{-4}	7.529×10^{-6}
Time — Quadratic ($Y = \beta_0 + \beta_1 x + \beta_2 x^2$)			
	β_0	β_1	β_2
β_0	2.381×10^{-2}	-1.062×10^{-3}	1.039×10^{-5}
β_1	-1.062×10^{-3}	8.352×10^{-5}	-1.039×10^{-6}
β_2	1.039×10^{-5}	-1.039×10^{-6}	1.443×10^{-8}
Time — Modified Gompertz ($Y = A\cdot exp\{-exp[(\mu max\cdot e/A)(\lambda-t)+1]\}$)			
	A	μmax	λ
A	2.354×10^{-3}	-3.788×10^{-4}	2.504×10^{-3}
μmax	-3.788×10^{-4}	4.566×10^{-4}	3.130×10^{-2}
λ	2.504×10^{-3}	3.130×10^{-2}	5.349×10^{0}
